# Spatially Resolved Multiomics: Data Analysis from Monoomics to Multiomics

**DOI:** 10.34133/bmef.0084

**Published:** 2024-01-13

**Authors:** Changxiang Huan, Jinze Li, Yingxue Li, Shasha Zhao, Qi Yang, Zhiqi Zhang, Chuanyu Li, Shuli Li, Zhen Guo, Jia Yao, Wei Zhang, Lianqun Zhou

**Affiliations:** ^1^ CAS Key Lab of Bio-Medical Diagnostics, Suzhou Institute of Biomedical Engineering and Technology, Chinese Academy of Sciences, Suzhou 215163, China.; ^2^ School of Biomedical Engineering (Suzhou), Division of Life Sciences and Medicine, University of Science and Technology of China, Hefei 230026, China.

## Abstract

Spatial monoomics has been recognized as a powerful tool for exploring life sciences. Recently, spatial multiomics has advanced considerably, which could contribute to clarifying many biological issues. Spatial monoomics techniques in epigenomics, genomics, transcriptomics, proteomics, and metabolomics can enhance our understanding of biological functions and cellular identities by simultaneously measuring tissue structures and biomolecule levels. Spatial monoomics technology has evolved from monoomics to spatial multiomics. Moreover, the spatial resolution, high-throughput detection capability, capture efficiency, and compatibility with various sample types of omics technology have considerably advanced. Despite the technological advances in this field, data analysis frameworks have stagnated. Current challenges include incomplete spatial monoomics data analysis pipeline, overly complex data analysis tasks, and few established spatial multiomics data analysis strategies. In this review, we systematically summarize recent developments of various spatial monoomics techniques and improvements in related data analysis pipeline. On the basis of the spatial multiomics technology, we propose a data integration strategy with cross-platform, cross-slice, and cross-modality. We summarize the potential applications of spatial monoomics technology, aiming to provide researchers and clinicians with a better understanding of how such applications have advanced. Spatial multiomics technology is expected to substantially impact biology and precision medicine through measurements of cellular tissue structures and the extraction of biomolecular features.

## Introduction

The human body is one of the most notable creations in nature. It consists of organs made of tens of thousands of cells that perform a variety of biological functions and coordinate various movements. Diseases arise from abnormalities within the cellular ecosystem and individual cell functions. Researchers have long focused on understanding cellular functions, aging, tissue development, and disease progression through comprehensive characterization of cellular compositions and intercellular interactions. Consequently, researchers at the National Institutes of Health have launched initiatives such as the Human Cell Atlas (HCA) and the Human Biomolecule Atlas Program (HuBMAP) to achieve these goals [1,2]. Despite important efforts, technological challenges still limit researchers’ ability to explore large datasets, multiple biomolecules, and high-resolution cellular maps simultaneously.

Breakthroughs in single-cell multiomics technologies have paved the way for the creation of high-resolution cellular maps. Single-cell multiomics technologies enable measurements of many individual cells within tissues and organs [3]. Nevertheless, the preparation of single-cell suspensions in experiments involves mechanical and enzymatic disruption of the original tissue architecture, resulting in a loss of the original spatial information for both the tissues and nuclei. To address these issues, researchers have introduced spatial monoomics technologies that enable biomolecule measurements.

Image-based spatial monomics is still confined to fewer than 100 targets but has achieved single-cell/subcellular spatial resolution. Spatial monoomics based on spatial barcodes have reached high-throughput goals, but it is difficult to achieve single-cell resolution spatially. Over time, spatial monoomics has been evolving toward single-cell resolution, high-throughput objectives, and compatibility with diverse sample types. Spatial monoomics techniques can simultaneously capture tens of thousands of transcripts and target hundreds of protein fluorescence signals within tissues [4]. If continuous spatial sampling and serial tissue sectioning techniques are applied, spatial monoomics technologies may evolve, leading to the development of 3-dimensional spatial monoomics (detecting entire organs or even organisms) and temporal spatial monoomics (multiple measurements over time) techniques, which would be powerful and exciting tools in biological research.

Spatial multiomics technology has been used to acquire 3 interrelated types of data: (a) image data, (b) biomolecular expression data, and (c) 2-dimensional coordinate system data. Single-cell omics computational models ignore spatial location information or process biomolecular expression data in isolation, which remains a challenge for traditional spatial monoomics technology. When omics reach single-cell or subcellular resolution, a region size that is too small would result in an insufficient number of gene detections per unit. The region size needs to encompass multiple cellular regions during the process of data analysis. Thus, Stereo-seq [5] provides a more refined granularity for cell annotation compared to Visium [6], which offers a coarser annotation, complicating the data integration from different technologies. Furthermore, using the most advanced technologies currently available requires several or dozens of sections to cover an entire tissue or organ. The vast amounts of high-resolution images, sequencing data, and tabular information, which differ in format protocols, urgently require high-performance computational methods. Therefore, the exponential increase in data volume, coupled with the lack of computational models and standardized approaches for new data types, has remained a considerable challenge in processing spatial multiomics data. There is a lack of format standardization among large datasets, high-resolution images, sequencing data, and tabular information. However, comprehensive techniques that involve preprocessing and analyzing data acquired by multiple omics technologies are lacking. In spatial multiomics, standardized analytical pipeline and integration strategies are currently lacking. This greatly limits the promotion and utilization of spatial multiomics technologies.

**Fig. 1.**
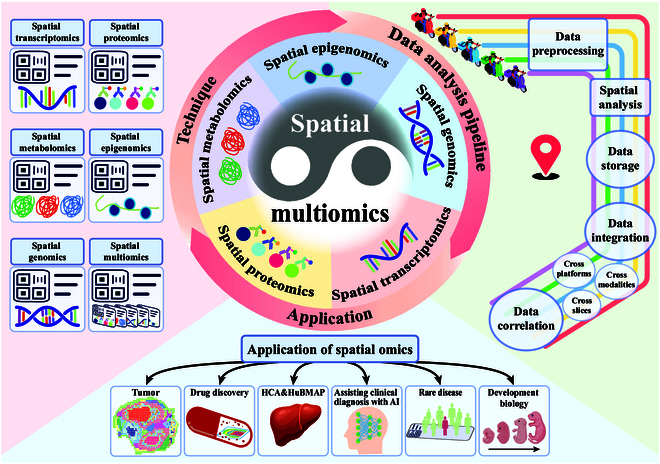
The frontier of transitioning from monoomics omics to multiomics: techniques, data analysis pipeline, and applications.

Therefore, in this paper, a spatial multiomics data integration strategy based on spatial monoomics technologies (with cross-platform, cross-slice, and cross-modality) is proposed. To address these challenges, complex algorithmic models applicable to various omics techniques have been designed. Additionally, the complete processes of different spatial monoomics technologies, data analysis pipeline (from preprocessing to downstream analysis), and application in life science are comprehensively summarized (Fig. 1).

## Spatial Monoomics Techniques

### Spatial transcriptomics

#### Image-based transcriptomics techniques

**Fig. 2.**
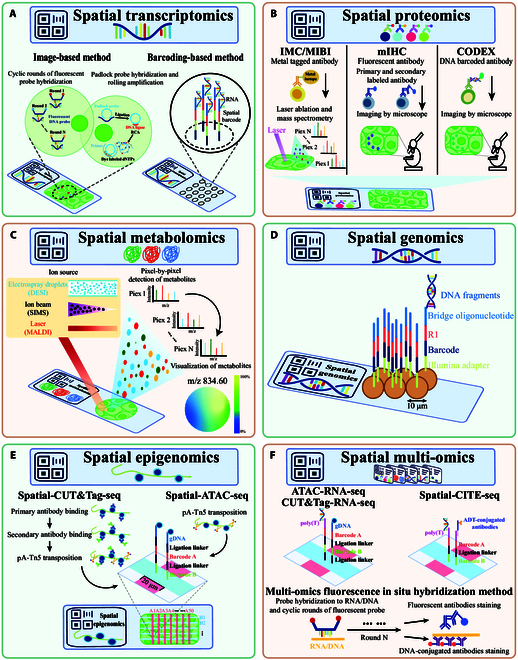
Schematic overview of spatial monoomics. (A) Spatial transcriptomics technologies can be divided into image-based and barcoding-based methods. ISH captures RNA molecules through cyclic rounds of fluorescent probe hybridization. The ISS generates sequencing products using padlock probe hybridization and RCA. The barcoding-based method uses mRNA-specific probes to nontargetly capture RNA molecules. (B) In spatial proteomics technologies, mIHC uses specific antibodies, which can include fluorescent antibodies or DNA barcode antibodies. Fluorescent antibodies are available as primary labeled antibodies and secondary labeled antibodies. (C) Spatial metabolomics technologies can be categorized based on the ion source type. Small-molecule metabolites are detected pixel by pixel, and their abundance information is projected onto tissue sections for visualization. (D) Spatial genomics: Slide-DNA-seq uses 10-μm barcoded bead arrays to capture spatially resolved genome-wide expression. (E) Spatial epigenomics: Spatial-CUT&Tag-seq and spatial-ATAC-seq employ dual microfluidic chips with probes to detect histone modifications and chromatin accessibility. Spatial-CUT&Tag-seq involves incubation with primary antibodies and secondary antibodies against epigenetic marks for DNA fragment tagmentation. Spatial-ATAC-seq uses a universal ligation linker to label accessible gDNA in situ. (F) Spatial multiomics: CUT&Tag-seq and ATAC-seq data were built by using a poly(T) DNA adapter to capture RNA molecules. Spatial-CITE-seq analyses RNA molecules and limited proteins using an ADT-conjugated antibody. DNA-seqFISH+ and DNA-MERFISH detect limited proteins using fluorescent or DNA-conjugated antibodies.

The first strategy for capturing transcripts in an organization is in situ hybridization (ISH) [7]. In ISH, fluorescently labeled DNA probes hybridize with RNA molecules in situ, allowing for quantitative RNA detection in tissues and cells via microscopy (Fig. 2A and Table 1). Femino et al. [8] used single-molecule fluorescence in situ hybridization (FISH) with oligonucleotide probes labeled with 5 fluorescent dyes to target mRNA. As the number of imaging rounds increases, the number of imaged genes increases exponentially. In addition, Lubeck et al. [9] introduced sequential FISH (seqFISH), which enables the detection of entire-genome transcripts using 4 to 5 fluorescent dyes and multiple rounds of hybridization; this technique was ultimately used to image 249 genes. However, this method has a potentially high error rate due to the nonspecific hybridization of the fluorescent signals, which accumulates with each hybridization. In the next-generation strategy, seqFISH+, the transcript encoding scheme, sequential detection process, and confocal microscopy platform were optimized. By using a panel of 60 pseudocolor signals and 3 imaging channels, seqFISH+ can obtain information on 24,000 genes after 3 rounds of hybridization [10]. Moreover, the multiplexed error-robust FISH (MERFISH) technique employs a binary coding strategy that is robust against detection errors, with each RNA assigned a unique N-bit binary code [11].

**Table 1.** Timeline of spatial monoomics techniques (2014 to 2020)

Technology	Years	Targets	Resolution	Sample (FF/FFPE)^a^	Ref
Spatial transcriptomics					
seqFISH	2014	249	Subcellular	FF	[9]
FISSEQ	2014	8,102	Subcellular	FF/FFPE	[12]
MERFISH	2015	140	Subcellular	FF	[11]
Visium	2016	>1,000	55 μm	FF/FFPE	[6]
seqFISH+	2019	24,000	Subcellular	FF	[10]
Slide-seq	2019	>1,000	10 μm	FF	[16]
DBiT-seq	2020	>1,000	10 μm	FF	[17]
ExSeq	2021	3,039	Subcellular	FF	[13]
Stereo-seq	2022	>1,000	0.22 μm	FF	[5]
Xenium	2022	313	Subcellular	FF/FFPE	[41]
VisiumHD	2024	>1,000	2 μm	FFPE	[15]
Spatial proteomics					
IMC	2014	40	1 μm	FF/FFPE	[18]
MIBI	2014	40	260 nm	FF/FFPE	[19]
t-CyCIF	2018	60	Single cell	FFPE	[21]
CODEX	2018	50	Single cell	FF/FFPE	[23]
IBEX	2022	38	Single cell	FFPE	[22]
Spatial metabolomics					
nanoDESI-MSI	2015	15 kDa	20 μm	Solid, frozen liquid	[26]
MALDI-MSI	2017	100 kDa	1.4 μm	Dried sample	[24]
SIMS-MSI	2020	3 kDa	20 nm	Dried sample	[25]
Spatial epigenomics					
Spatial-CUT&Tag-seq	2022	>1,000	20 μm	FF	[27]
Spatial-ATAC-seq	2022	>1,000	20 μm	FF	[29]
Spatial genomics					
Slide-DNA-seq	2022	>1,000	10 μm	FFPE	[31]

^a^ The sample types mainly include FF (fresh frozen) and FFPE (formalin fixation and paraffin embedding).

Another image-based spatial transcriptomics sequencing technology is in situ sequencing (ISS). The ISS technique involves in situ reverse transcription of RNA into cDNA within fixed cells or tissues. Rolling circle amplification (RCA) is then used to amplify the signal, producing RCA products. Transcript information is obtained through second-generation sequencing technology known as sequencing by ligation (Fig. 2A). Fluorescence in situ sequencing (FISSEQ) technology is used to convert mRNA into cDNA via reverse transcription primers, followed by RCA and sequencing by oligonucleotide ligation and detection to retrieve transcriptome information [12]. Compared with ISS, FISSEQ is a nontargeted sequencing method with low sensitivity due to inefficient in situ reverse transcription–polymerase chain reaction (RT-PCR) and sequencing. In addition, expansion sequencing (ExSeq) technology distinctively uses short ISSs as spatial barcodes, which, when remapped with full-length transcriptome information, can be used to effectively acquire spatial positional information of the entire transcriptome [13]. Furthermore, the method developed by Xenium [14] enables the simultaneous detection of high-throughput transcripts and low-throughput proteins at single-cell resolution.

Image-based spatial transcriptomics techniques have subcellular resolution, revealing diverse cellular states. However, these techniques rely on designing targeted gene panels, limiting their ability to capture complete transcriptomes and thus the discovery of rare biological features.

#### Spatial barcoding-based transcriptomics techniques

Spatial transcriptomics technology allows unbiased capture of intact transcripts within tissues with spatial barcodes (Fig. 2A and Table 1). Ståhl et al. [6] achieved this through spatial transcriptomics technology, where mRNA-specific capture probes with spatial barcodes are fixed on the surface of a slice, enabling the matching of sequencing information with spatial location data after high-throughput sequencing. This technology was later successfully commercialized as a Visium platform. This technique has a spatial resolution of 55 μm within a 6.5 mm × 6.5 mm area. The next-generation strategy, VisiumHD, has a spatial resolution of 2 μm [15]. To further improve the spatial resolution, Rodriques et al. [16] developed Slide-seq technology, which uses encoded microspheres instead of a spatial transcriptomics spatial array. In Slide-seq, barcodes, biomolecule encodings, and capture sequences are placed on encoded microspheres; this technique achieves single-cell spatial resolution with a capture microsphere diameter of 10 μm. Spatiotemporally enhanced resolution omics sequencing (Stereo-seq) was used to develop a DNA nanoball (DNB) sequencing chip, further increasing the spatial resolution to 220 nm and enabling analysis of tissue areas up to 42.25 cm^2^, outperforming other techniques [5]. Spatial transcriptomics technology has evolved beyond the development of encoding techniques toward chip design and data integration with increasing spatial resolution. The deterministic barcoding in tissue sequencing (DBiT-seq) technique innovatively employs microfluidics to label tissue regions in situ via spatial encoding methods, enabling joint detection of transcriptomes and proteomes. The DBiT-seq technique utilizes 50 parallel channels (A1 to A50) and 50 vertical channels (B1 to B50), with 2,500 combinations of A*i*B*i* (*i* = 1 to 50), but the channel span is limited to 20 μm; thus, single-cell spatial resolution cannot be achieved with this technique [17].

### Spatial proteomics techniques

There are 2 main types of spatial proteomics technologies for detecting specific nucleotides: mass cytometry (MC) and multiplex immunohistochemistry (mIHC) (Fig. 2B and Table 1).

MC methods primarily include imaging mass cytometry (IMC) and multiplexed ion beam imaging (MIBI) [18,19]. The principle behind MC involves each antibody being conjugated to a different metal by chelating polymers (Fig. 2B). During imaging, a laser ablates the tissue pixel by pixel, and a mass spectrometer is used to quantify the ions released at each pixel. These data are used to infer the presence and spatial distribution of specific protein antigens in the tissue. The advantage of this technology is its high signal-to-noise ratio, but the metals used are rare and expensive, and the number of targets is limited to between 10 and 50.

Another technique, mIHC, is based on tyramide signal amplification (Fig. 2B) [20]. This technique involves binding different fluorescent groups to specific antibodies, allowing for multiple sequential immunostainings of a single cell or tissue sample. Variants of these techniques with different oligonucleotide probes have been developed. The first method uses directly labeled fluorescent primary or secondary antibodies. Direct methods include t-CyCIF and IBEX [21,22]. To reduce autofluorescence and nonspecific antibody binding, different strategies are used by each method. IBEX uses lithium borohydrate to eliminate fluorescence signals from various fluorescently conjugated antibodies. t-CyCIF involves the incubation of samples with secondary antibodies followed by fluorophore oxidation in a high pH hydrogen peroxide solution under light; this process is known as fluorophore bleaching. Other fluorescent cyclic methods are based on DNA barcoding of antibodies (Fig. 2B). The CODEX technique allows for the simultaneous detection and analysis of more than 50 protein markers by binding the required fluorescent dyes to oligonucleotide sequences that are complementary to the barcode, thus overcoming the limitations of the number of fluorescent imaging channels in the visible spectrum [23].

However, current spatial proteomics technologies are expensive because of the use of multiple antibody techniques. Furthermore, the reduced throughput of targets and the design of targeted antibody panels remain challenges in capturing comprehensive proteomic information.

### Spatial metabolomics techniques

Spatial metabolomics techniques combine mass spectrometry imaging (MSI) with metabolomic techniques, enabling pixel-by-pixel detection of endogenous metabolites and exogenous drugs in tissue sections and the collection of spatial location information. MSI technology can be divided into different categories depending on the ion source: matrix-assisted laser desorption/ionization mass spectrometry imaging (MALDI-MSI), secondary ion mass spectrometry imaging (SIMS-MSI), and desorption electrospray ionization mass spectrometry imaging (DESI-MSI) (Fig. 2C and Table 1).

MALDI-MSI involves mixing a test material with a matrix biomolecule that can absorb ultraviolet (UV) light at 337 or 355 nm to form a cocrystal [24]. After ionization, the sample weight can be obtained by detecting the *m*/*z* information of different fragments. The spatial resolution of this technique can reach 1.4 μm. The SIMS-MSI method has the highest spatial resolution among the MSI techniques, with dynamic SIMS approaches having ultrahigh resolutions of 20 to 50 nm. Depending on the type and size of the ion beam, SIMS techniques can be divided into dynamic and static SIMS methods. Time-of-flight SIMS imaging is commonly used in life science research [25]. However, these imaging technologies all require a vacuum, whereas nanoDESI-MSI can ionize and image test molecules under normal atmospheric pressure with a spatial resolution of 20 μm [26]. In conclusion, spatial metabolomics technology has enabled spatial visualization and analysis of various metabolites, greatly facilitating research on biomolecule histology and pathology.

### Spatial epigenomics techniques

Gene expression and function considerably impact cell behavior. Epigenomic modifications, which act as regulatory elements, can influence gene expression and thereby cellular behavior during development, tissue evolution, and environmental responses. Spatial genomics and spatial epigenomics both employ barcode-based methods to capture DNA segment (Fig. 2D and E and Table 1).

Deng et al.[27] developed spatial cleavage under targets and tagmentation (Spatial-CUT&Tag), which utilizes the pA-Tn5 transposome and next-generation sequencing (NGS) technology to analyze whole-genome protein modification maps at high spatial resolution in frozen tissue sections (Fig. 2E).

While some methods use spatial barcoding and in situ image capture techniques, the CUT&Tag technique uses microfluidic encoding. First, an antibody specific to the target histone modification is added to the microfluidic channel. Subsequently, a secondary antibody is introduced to activate the cutting activity of the pA-Tn5 enzyme. After activating the transposome, adapters containing a ligation linker are inserted into the genomic DNA at the sites recognized by the histone mark antibody. Finally, DNA is extracted, amplified by PCR to construct a library, and subjected to high-throughput sequencing. Compared with chromatin immunoprecipitation technology, CUT&Tag does not require NGS library preparation and has a higher signal-to-noise ratio, significantly simplifying the experimental process and improving reproducibility [28].

Deng et al. [29] introduced another novel technology, spatially resolved sequencing of chromatin accessibility at the whole-genome scale (Spatial-ATAC-seq, Fig. 2E). This method combines 2-dimensional coding of tissue spatial information using microfluidic technology and ATAC-seq to analyze chromatin accessibility at a genome-wide scale. The library construction process in each microfluidic channel is as follows. First, Tn5 transposition was carried out in a fixed tissue section, and adapters containing a ligation linker were inserted into accessible genomic loci. Tn5 transposition cut DNA fragments that are then amplified by PCR to prepare the library. Furthermore, comparisons between the Spatial-ATAC-seq results (at different resolutions) and the single-cell ATAC-seq results indicate that the data quality of both techniques can reach the same level.

### Spatial genomics techniques

Bouwman et al. [30] defined spatial genomics as the study of how the genome sequence or spatial position of the genome in the nucleus varies among cells and different cell types at defined locations in tissues or organs in multicellular organisms. Currently, spatial genomics techniques are in the early stages of application. In the Slide-DNA-seq [31] technique, a 3-mm spatial index array with polystyrene magnetic beads attached to the surface is used (Fig. 2D). The beads, which carry spatial barcodes, capture DNA fragments from tissue sections in an unbiased manner. This method efficiently obtains spatial location and abundance information for DNA fragments through spatial barcoding and paired-end sequencing. Additionally, 10-μm-thick beads are sufficient for realizing single-cell resolution. The slide-DNA-seq analysis workflow can be used to locate target areas with a genomic resolution of approximately 1 Mb, thereby enabling the simultaneous mapping of the spatial locations of different DNA mutation features.

The development of Slide-DNA-seq fills a knowledge gap in spatial genomics, suggesting new directions for future technological innovations. These methods increase our ability to understand and target complex genomic architectures and interactions that contribute to tumor heterogeneity and challenges in cancer treatment.

### Spatial multiomics techniques

The use of 2 microfluidics chips enabled the development of a versatile spatial multiomics framework based on spatial barcodes. In Group A, universal adapters are used to attach barcode A to gDNA or poly(T)-cDNA, and Group B is connected to Group A, forming a 2-dimensional coordinate system (Fig. 2F). Streptavidin-coated magnetic beads are used to enrich cDNA, with gDNA remaining in suspension, followed by NGS library construction and sequencing. In the spatial ATAC-RNA-seq technique, universal adapters are used to label transposase-accessible genomic DNA loci in situ [32]. The Spatial-CUT&Tag-RNA-seq technique involves incubation with primary antibodies against epigenetic markers to assess histone modifications [32]. The same chips can also be incubated with biotinylated DNA adaptors to initiate reverse transcription for transcript capture. Combining NGS technology with microscopic imaging enables the joint mapping of the epigenome and transcriptome at different spatial locations. However, the spatial resolution of this technology is limited to regional levels (20 μm pixel size).

In addition, DNA-seqFISH+ [33] and DNA-MERFISH [34], modifications of seqFISH and MERFISH technologies, respectively, enable direct imaging of intracellular DNA loci, chromosomal and nuclear structures, and transcripts using fluorescent antibodies or primary antibodies conjugated with DNA oligonucleotides detectable by fluorescently labeled readout probes (Fig. 2F). DNA-MERFISH, which uses immunofluorescent antibodies to label nuclear structures, can detect more than 1,000 genomic loci and more than 1,100 transcripts for sequencing [34]. Although DNA-seqFISH+ can detect only 70 mRNAs, it can detect thousands of genomic loci, transcriptionally active sites, and 17 antibodies when labeling nuclear structures [33].

**Fig. 3.**
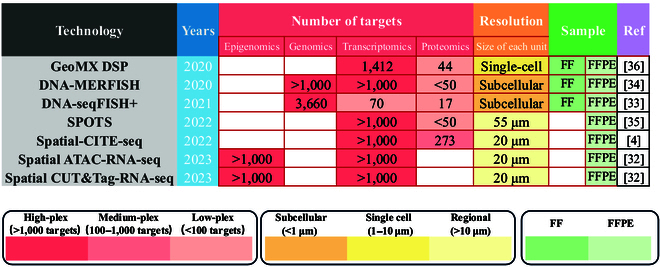
Timeline of the spatial multiomics techniques.

Spatial multiomics techniques that combine transcriptomics and proteomics often have low spatial resolution and low protein throughput. Spatial protein and transcriptome sequencing has improved upon Visium technology in that it uses antibody-derived tags (ADTs) to detect more than 30 protein markers [35]. With the NanoString GeoMx Digital Spatial Profiling (DSP) technique, genes or proteins with RNA probes or antibodies labeled with special oligo sequences can be quantified [36]. UV irradiation of regions of interest (ROIs) leads to the release and capture DSP barcodes on probes/antibodies for NGS. The targeted whole-transcriptome DSP assay can map more than 150 proteins and 18,000 transcripts simultaneously. However, this technology has yet to advance beyond single-cell resolution, and only small ROIs can be analyzed. Spatial-CITE-seq and Spatial-CUT&Tag-RNA-seq are similar methodologies that use spatial barcodes with poly(T) DNA adaptors or ADT-conjugated antibodies in the 2-dimensional coordinate system formed by Group A and Group B to achieve joint imaging of proteins and transcripts (Fig. 2F) [4]. Spatial-CITE-seq can map 200 to 300 proteins in tissue at 20 μm resolution [4]. Figure 3 summarizes the recent advancements in the aforementioned multiomics technologies concerning target numbers, spatial resolution, and sample applicability.

## Data Analysis Pipeline for Spatial Monoomics

**Fig. 4.**
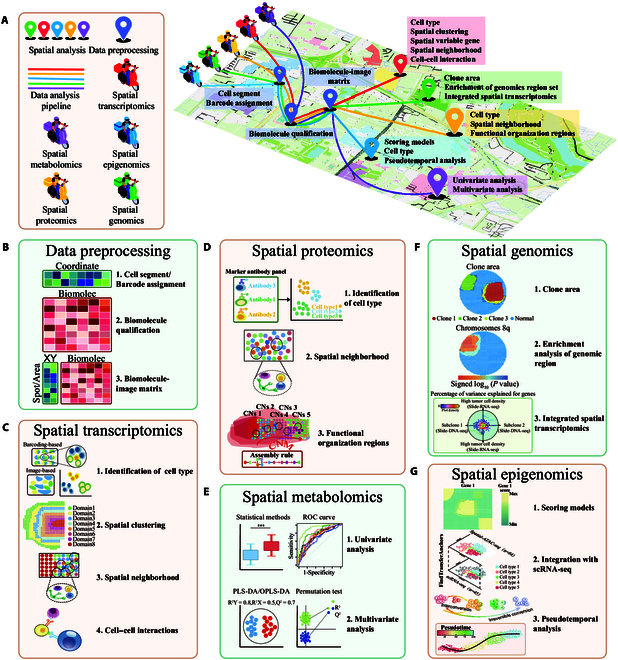
Spatial monoomics data analysis pipeline. (A) The data analysis pipeline summarizes the complete process for spatial monoomics. (B) Data preprocessing involves creating a matrix by merging spatial location information with biomolecular quantitative data. (C) Barcode-based and image-based technologies use different strategies to identify cell types. Clustering analysis explore spatial regions with unique gene expression patterns. Spatial neighborhood analysis detects cell communication through gap junctions by identifying neighboring cell types. Cell–cell interactions were calculated based on ligand–receptor gene expression values. (D) Cell types are annotated using a marker panel of protein antibodies. Spatial neighborhood analysis detects pairwise cell–cell interaction. Cellular neighborhoods form functional cell units. (E) Raw data were processed through OPLS-DA or PLS-DA to construct classification models. Metabolite markers were subsequently screened via multivariate analysis. (F) Distinct clone areas were identified. Genomic region enrichment analysis revealed a greater number of genetic aberrations within tissue regions. By integrating Slide-RNA-seq, a series of subclone/tumor density-associated genes were identified. (G) Scoring models can be used to evaluate the correlation with target gene datasets to detect biological functions. Cell types were identified through deconvolution via scRNA-seq, and pseudotemporal analysis was used to assess cell differentiation pathways.

The complexity of spatial monoomics data makes these data both powerful and difficult to interpret. These data are typically collected in batches and generated in large quantities. The large amount of data available poses great challenges for computational biologists, leading to difficulty in data analysis and the construction of computational pipelines. Here, we review recent computational methods in spatial transcriptomics, spatial proteomics, spatial metabolomics, spatial epigenomics, and spatial genomics (Fig. 4A and Table 2).

**Table 2.** Summary of the computational methods applied in spatial monoomics

Category	Usage	Package	Environment	URL	Ref
Spatial transcriptomics	Identification of cell types	Tangram	Python	https://github.com/broadinstitute/Tangram	[41]
		CellTrek	R	https://github.com/navinlabcode/CellTrek	[42]
		SPOTlight	R	https://github.com/MarcElosua/SPOTlight	[43]
		Cell2location	Python	https://github.com/BayraktarLab/cell2location	[44]
		RCTD	R	https://github.com/vigneshshanmug/RCTD	[45]
	Spatial clustering	BayesSpace	R	https://github.com/edward130603/BayesSpace	[47]
		StLearn	Python	https://stlearn.readthedocs.io/en/latest/	[48]
		SpaGCN	Python	https://github.com/jianhuupenn/SpaGCN	[49]
	Spatial neighborhood	HistoCAT	MATLAB	https://schapirolabor.github.io/histoCAT/	[53]
		CytoMap	MATLAB	https://github.com/DrStoltzfus/CytoMAP	[54]
		CytoCommunity	R, Python	https://github.com/huBioinfo/CytoCommunity	[55]
	Cell-cell interactions	SpaOTsc	Python	https://github.com/zcang/SpaOTsc	[56]
		SpaTalk	R	https://github.com/ZJUFanLab/SpaTalk	[57]
		COMMOT	Python	https://github.com/zcang/COMMOT	[58]
Spatial proteomics	Identification of cell types	PhenoDisco	R	https://www.rdocumentation.org/packages/pRoloc/versions/1.12.4/topics/phenoDisco	[66]
		PhenoGraph	Python	https://github.com/jacoblevine/PhenoGraph	[67]
		MAP	Python	http://bioinfo.sibs.ac.cn/shaolab/MAP	[68]
	Spatial neighborhood	Cytomap	MATLAB	https://github.com/DrStoltzfus/CytoMAP	[54]
		HistoCAT	MATLAB	https://schapirolabor.github.io/histoCAT/	[53]
		SpatialLDA	Python	https://github.com/calico/spatial_lda	[69]
	Functional organization regions	TissueSchematics	Python	https://github.com/nolanlab/TissueSchematics/	[71]
Spatial metabolomics	Univariate analysis	scikit-learn	Python	https://scikit-learn.org/stable/index.html	[84]
		SciPy	Python	https://scipy.org	[80]
	Multivariate analysis	scikit-learn	Python	https://scikit-learn.org/stable/index.html	[84]
Spatial genomics	(a) Clone area	slide-DNA-seq analysis	MATLAB, Python	https://github.com/buenrostrolab/slide_dna_seq_analysis	[45]
	(b) Enrichment of genomic region set				
	(c) Integration with spatial transcriptomics				
Spatial epigenomics	(a) Scoring models	Spatial epigenomics analysis	R	https://zenodo.org/record/5797109#.YenP5-rMKUk	[27]
	(b) Integration with scRNA-seq		R	https://github.com/dyxmvp/Spatial_ATAC-seq	[29]
	(c) Pseudotemporal analysis				

### Data analysis for spatial transcriptomics methods

#### Data preprocessing

When applying spatial transcriptomics techniques, the first task that must be performed is the preprocessing of raw data. Upstream preprocessing involves converting data files into gene–spot matrix objects, facilitating data visualization and downstream analysis (Fig. 4B).

Image-based spatial transcriptomics techniques use hybridization probes targeted for ISH and ISS, with each RNA biomolecule having a unique binary barcode. Consequently, processing image data has become an important task in image-based spatial transcriptomics. The conversion of fluorescence images into a multidimensional matrix of gene–cell images involves several steps. Initially, noise and clutter unrelated to the input image must be filtered out. Images from multiple hybridization rounds are subsequently aligned to construct a 2-dimensional coordinate system. Finally, the fluorescence signals are read as “1” and “0” and combined into barcodes and sequences. These values are then used to obtain the transcript abundance for each pixel, and unmatched signals are discarded. Finally, cell segmentation is performed by identifying potential cell regions in the image. The obtained transcript abundance is matched with that of the cells to ultimately construct a gene–cell–image matrix.

Unlike image-based spatial transcriptomics methods, which require image segmentation to obtain each cell’s coordinate data, spatial barcode-based spatial transcriptomics methods collect the coordinate data for each barcode through the sequencing chip. Consequently, data preprocessing for spatial barcode-based transcriptomics involves the following steps. First, after the tissue image is registered with the sequencing chip image, each sequence’s barcode corresponds to a coordinate index provided by the sequencing chip. Next, low-quality sequences and common sequencing adapters are removed via Trimmomatic software [37]. The captured sequences are subsequently aligned and annotated with reference genes via STAR [38] software to determine the transcript abundance at each location; finally, the gene–spot coordinate matrix is generated.

#### Identification of cell types

The identification of cell types is a fundamental step in using spatial transcriptomics technology to map organ atlases and explore the spatial heterogeneity of diseases. As spatial transcriptomics technology cannot simultaneously have single-cell spatial resolution and high transcript throughput, various cell-type identification strategies are adopted for different spatial transcriptomics technologies. For imaging-based spatial transcriptomics techniques, 2 strategies are employed: (a) cell identification on the basis of features and (b) mapping in conjunction with single-cell RNA sequencing (scRNA-seq). Spatial barcoding-based spatial transcriptomics methods use deconvolution in conjunction with scRNA-seq (Fig. 4C).

The cell identification results of imaging-based spatial transcriptomics techniques with single-cell or even subcellular resolution are similar to those of well-established single-cell transcriptomic methods (Fig. 4C). After principal component analysis (PCA) of the matrix using spatial transcriptomic analysis toolkits such as Seurat [39] and Giotto [40], traditional Leiden or Louvain clustering is applied to identify clusters of cells with similar gene expression patterns. Biologists manually annotate cell clusters on the basis of prior knowledge and marker genes. Although this process may seem straightforward, marker genes are not necessarily highly expressed in specific cell clusters, and pathological changes or cell cycle factors can reverse marker gene expression patterns. To address unreasonable annotation standards, researchers have developed various approaches to map spatial transcriptomic data directly via scRNA-seq, which has high resolution and sensitivity. Tangram [41] inferred the probability of a cell in a tissue being a certain type via “pseudospace” data. CellTrek embeds single-cell transcriptomic and spatial transcriptomic data into the same dimension, mapping the position information of all cells on spatial slices [42]. Although most methods obtain results that are consistent with real-world data, prior biological knowledge is still needed.

Spatial transcriptomics techniques based on spatial barcoding typically do not have single-cell resolution, and the gene–spot matrices obtained through such methods are usually used for downstream data analysis. Therefore, direct clustering of the matrix to obtain the results of different cell clusters is not due to differences in gene expression patterns but rather to differences in the composition of different cell types. Deconvolution algorithms provide a solution to this problem by jointly inferring the composition of different cell types at each location with scRNA-seq quantification (Fig. 4C). Commonly used algorithms include SPOTlight [43] (using nonnegative matrix factorization regression), Tangram [41] (using deep learning), Cell2location [44] (using a negative binomial distribution), and RCTD [45] (using nonnegative least squares regression). Li et al. [46] developed 18 benchmark methods and suggested that Cell2location, RCTD, and SPOTlight should be used as the primary tools in related research.

#### Spatial clustering

Once individual cell types or the composition of different cell types at each location are successfully identified, downstream analysis techniques are used to explore the collective properties of the clustered cells. Since most commercial spatial transcriptomic technologies do not achieve single-cell resolution, resulting in multiple cell types at each location, the clustering results of the gene–spot matrix are specific to the cellular spatial domain (Fig. 4C).

Biomolecule clustering is performed with the commonly used clustering methods in traditional scRNA-seq data analysis, such as hierarchical, K-means, Louvain, Leiden, and k-nearest neighbor (KNN) methods. Biomolecule clustering is performed on the basis of the correlations among gene expression profiles within cells or spots, and spatial and tissue information are not considered. BayesSpace [47] uses Bayesian methods to model gene expression matrices and weight spatially neighboring spots to achieve spatial clustering. In stLearn [48], the gene matrix for clustering is adjusted on the basis of spatial morphological gene expression data, with features extracted from tissue images and gene expression patterns of adjacent spots. In SpaGCN [49], spatial position and histological information are considered, and unsupervised clustering is performed based on gene expression matrices aggregated from adjacent locations via graph convolutional layers. Introducing spatial information in clustering strategies may lead to excessive spatial influence, resulting in block-like clusters in space deviating from the true biological structure. In a benchmark comparison of cell clustering methods, Seurat-Louvain and SpaGCN exhibited the best accuracy among 15 clustering methods [50]. Methods that incorporate additional spatial location and histological image information for clustering do not necessarily outperform methods that use only gene expression information.

#### Spatial neighborhood

Cells communicate with their microenvironment in multiple ways, including the release of soluble molecules and direct cell-to-cell contact, actively altering their transcriptome in response to external signals [51]. To better understand critical biological factors such as disease and development, understanding the various ways in which cells communicate with each other is essential. In addition to using ligands and receptors for communication, cells utilize various channels, including gap junctions, to communicate with neighboring cells (Fig. 4C) [52]. Therefore, adjacent cells may form a functional unit that synergistically performs specific biological functions in the microenvironment. One strategy to characterize functional units in the microenvironment is to identify cell types that may be enriched in adjacent regions and locate spatial areas containing functional units (Fig. 4C). HistoCAT [53] was an important computational method developed for analyzing cell neighborhoods. This method uses basic statistical techniques to identify the most significant cell phenotypes that interact with one another. The identified interactions are then visualized via heatmaps, which highlight the importance of interactions between each pair of phenotypes. CytoMap [54] supports user-defined partitioning of local cell neighborhoods. It transmits information such as cell composition types, density, and transcripts of different neighborhoods to a self-organizing map model for clustering and visualization of functional units. In CytoCommunity [55], graph-based pooling methods are utilized to identify new, condition-specific cell functional units via both scRNA-seq and spatial transcriptomics under the supervision of sample labels. Cell neighborhood analysis enables researchers to discover spatially specific functional units. Future work should focus on the mechanisms underlying interactions within or between units and the microenvironment.

#### Cell–cell interactions

Cells coordinate with each other within the microenvironment to execute specific biological functions. This mode of operation is known as cell–cell interaction or cell–cell communication, with cells migrating to specific regions and communicating with neighboring cells through signaling molecules. This mode of operation is achieved through autocrine, paracrine, endocrine, and juxtacrine mechanisms [52]. Among these pathways, endocrine signaling requires molecules to enter the circulatory system to travel longer distances, and spatial transcriptomics is not suitable for analyzing this process.

The main components of bioinformatics tools are ligand–receptor (LR) databases and computational models that calculate the likelihood of cell–cell interactions on the basis of gene expression values (Fig. 4C). In SpaOTsc [56], a spatial metric is constructed by utilizing gene expression similarities from scRNA-seq and spatial transcriptomics data, as well as spatial distances between cells. However, SpaOTsc can be applied only on spatial transcriptomics platforms with single-cell resolution. To address this difficulty, in SpaTalk [57], the LR proximity distance and the principle of ligand–receptor–target coexpression are integrated to model and infer cell–cell interactions mediated by LR interactions for techniques without single-cell resolution. Additionally, there are competitive relationships between different types of ligands and receptors. COMMOT [58] handles complex biomolecule interactions and constructs cell–cell interaction networks through optimal transport modeling.

### Data analysis for spatial proteomics methods

#### Data preprocessing

Spatial proteomics methods can be divided into mass spectrometry-based proteomics preprocessing methods and image-based proteomics preprocessing methods. In addition to baseline correction, smoothing, denoising, peak extraction, and peak alignment (as discussed in the context of spatial metabolomics), mass spectrometry-based proteomics techniques require preprocessing steps that are similar to those used by image-based spatial proteomics methods, including image preprocessing, cell segmentation, missing value imputation, and normalization.

Owing to the inherent limitations of microscope systems, cyclic images often exhibit blurring, multidirectional drift, and misalignment. Additionally, endogenous autofluorescence in tissues generates background noise. Therefore, efficient software is required to process large-scale imaging data [59]. To conduct qualitative and quantitative statistical analyses of proteins/peptides, it is necessary to perform feature extraction on each segmented cell. Statistical analysis techniques generate raw quantitative protein matrices, where each cell is represented as a separate vector, and each cell’s characteristics are described (Fig. 4B). These features include signal intensity, shape, and spatial coordinates, among others [60]. The cell features obtained are crucial for subsequent cell annotation, organelle localization, and functional identification.

The obtained protein–cell matrix must be preprocessed, which primarily includes the imputation of missing values and data normalization. Low-abundance proteins that are missing need to be addressed to prevent bias in subsequent data analysis. Missing values can be imputed by interpolating incomplete time series data and filling in missing data on the basis of fitted values. Machine learning (ML) methods such as KNN can also be used to impute missing values [61]. Overlapping nonbiological factors can introduce biases in mass spectrometry data. Data normalization techniques can be applied to represent each protein on the basis of the sum, average, or median values in each sample/channel [62]. When different cell types are present, the weighted trimmed means of M-value normalization and log expression normalization, as determined through the edgeR and DESeq2 packages, are appropriate [63,64]. PCA can be applied to assess the quality of the data after normalization.

#### Identification of cell types

Spatial proteomics techniques use a similar cell-type annotation process to transcriptomics methods, which generally includes clustering and annotation of cell clusters (Fig. 4D). Designing spatial proteomics experiments requires setting up a panel of protein antibodies for detection in advance, with various cell-type marker proteins selected for identifying cell types. In spatial proteomics, k-means and hierarchical clustering methods are commonly used for clustering and analysis of different cell types. In k-means clustering, data are divided into k distinct cell clusters; however, this approach focuses on the generation of clusters while ignoring the relationships between them [65]. Hierarchical clustering techniques require complex parameter adjustment to ensure consistency with real-world results. Semisupervised ML techniques can be used for proteomics clustering analysis. PhenoDisco [66], which is based on the Gaussian mixture model, can be used to identify potential organelle groups in mass spectrometry-based proteomics data. PhenoGraph [67] relies on a graph/network of recorded events and their connections to accurately cluster events into phenotypic categories.

Immunofluorescence imaging annotation methods rely heavily on pathological expertise and are limited by a small number of channels and reliance on a few marker proteins. Additionally, immunofluorescence imaging annotation methods are subjective and time-consuming. Various automated annotation software programs have been developed to address these shortcomings. In MAP [68], a feedforward neural network is used, significantly improving computational efficiency and enabling rapid and accurate cell-type annotation. If a cell does not match predefined cell types, it is considered the same type as its neighboring cells. However, this technique requires high-quality imaging data.

#### Spatial neighborhood

Cells need to cooperate with each other to perform biological functions. Therefore, different cell types migrate to specific spatial locations and form repetitive functional units within tissue areas (Fig. 4D). Signal molecules regulate cells not only by altering gene expression but also by triggering corresponding responses to proteins, such as downstream molecules. In Cytomap [54], users can set the number and distance of adjacent cells; however, this approach is very subjective. histoCAT [53] is also widely applicable to spatial proteomics. Spatial-LDA [69] is another tool suitable for analyzing cell neighborhoods in spatial proteomics, but it has lower computational efficiency than other approaches. Direct contact between cells may indicate interactions between 2 cell types. The interaction between cells can be inferred on the basis of the frequency of cell-to-cell contact. Goltsev used the log odds ratio to measure the specificity of interactions between 2 cell types in mouse spleens. Positive interactions between macrophages and erythroid cells were observed in erythroblast islands in mouse spleens, whereas in the white pulp, homotypic cell associations played major roles [70]. There are still marked limitations in pairwise cell–cell interaction analysis. Specifically, existing methods rely on the enrichment of adjacent cells around the central cell to assess interactions, resulting in most cell–cell interactions involving cells of the same type. The introduction of ligand–receptor databases or additional targeted signaling biomolecule information could correct enrichment results in the future.

#### Functional organization regions

The previous sections discuss how cellular neighborhoods (CNs), composed of various cell types, form functional units, whereas functional organization regions involve functional units assembling into higher-level and more complex organizational areas (Fig. 4D). In these areas, complex organizational functions and cellular evolutionary processes are performed. Bhate et al. [71] proposed tissue schematics, an algorithmic model that divides organs into 3 parts: CNs, CN-combination maps (CNMs), and assembly rule (AR) maps (Fig. 4D). CNs form CNMs under the control of ARs. This model and the CODEX dataset have been utilized to study the functional organization regions in human lymphoid tissue (HLT). Eleven types of CNs were identified as the basic functional units of HLT, with each cell type uniformly distributed within the CNs. The CN composition of each CNM structurally matched known immune structures, demonstrating that interfaces are sites of cellular interactions. At local interfaces, interactions between different CNs (BTCN-TCN interfaces) can be used to map the composition of different cell types. The ARs for CNs from different HLTs vary, and the immune-tumor microenvironment (TME) of the human colorectal cancer (CRC) dataset was used for validation.

### Data analysis for spatial metabolomics methods

#### Data preprocessing

In MSI, preprocessing methods include baseline correction, smoothing, denoising, peak extraction, peak alignment, normalization, and biomolecule annotation.

Anomalous peaks occur during MSI experiments due to systemic instrument issues, leading to high-frequency noise and a broad chemical background. Two techniques, total variation minimization and the Chambolle algorithm, can be used for edge-preserving denoising of *m*/*z* images [72]. These methods help maintain the integrity of the edges while removing noise, effectively clarifying the *m*/*z* image outputs. The goal of peak extraction in MSI is to consolidate the mass spectrometric signals of a compound into a single peak, thus simplifying the mass spectrum into a list of signal peaks. Lieb et al. [73] developed a peak extraction algorithm for MALDI-MSI that robustly utilizes spatial information, with strong performance in peak extraction, baseline correction, and denoising. When a peak list is constructed from spectral peak values associated with a dataset, the *m*/*z* values selected for a peak may vary slightly among different samples, leading to difficulty in understanding biological differences among samples. The global intensity-guided peak matching and alignment (GIPMA) algorithm [74] maximizes the number of valid peak clusters by adaptively and intelligently selecting suitable chemical shift tolerances, thereby maximizing the alignment of homogeneous signal peaks across samples. Since MSI data are acquired from different samples, the dataset contains many mass spectra. To reduce differences in the abundance of metabolites in different mass spectra, it is common to compare and calibrate the *m*/*z* values of the same ions, uniformly scaling their abundance values. The most commonly used normalization method is the total ion count (TIC), in which the abundance values of all biomolecule ions are divided by the total number of ions. Specific tools used for annotation include METASPACE [75].

#### Univariate analysis

Univariate statistical analysis methods are commonly employed to compare differences in peak values across samples, aiming to identify specific biological markers. While *t* tests and analysis of variance (ANOVA) are the most frequently used statistical methods [76,77], nonparametric tests that do not assume a Gaussian data distribution, such as the Mann–Whitney *U* test and Kruskal–Wallis test, are also utilized for interpreting MSI data (Fig. 4E) [78,79]. The SciPy package includes a variety of analytical tools [80]. On the basis of the results of these statistical tests, volcano plots can be used to visualize ions with statistically significant intensity differences between groups. If the data distribution is non-Gaussian, nonparametric tests such as the Mann–Whitney *U* test, which assumes similar distribution shapes but different locations on the basis of rank sums, can be applied. The Kruskal–Wallis test is also used for non-Gaussian distributed data across multiple categories, and its *P* value interpretation is similar to that of ANOVA. The appropriate statistical method depends on the fundamental properties of the data. The most straightforward approach is to examine the fold change between groups. The fold change is the ratio of the average intensity of the selected ions in the test group to that in the control group. When multiple tests are conducted, the false-positive rate may be significantly increased. The goal of biomarker discovery research is to correctly classify samples. The receiver operating characteristic (ROC) curve is typically used for biomarker evaluation [81]. The area under the curve (AUC) indicates the predictive score and is used to evaluate the classification effectiveness of potential biomarkers.

#### Multivariate analysis

PCA is an unsupervised metabolomic analysis technique used to reduce the dimensionality of MSI data by projecting data to a lower-dimensional space. PCA is primarily used to observe differences among samples. Each sample is analyzed to identify small biomolecule metabolite markers, with each marker representing a dimension. Therefore, the original data are complex and multidimensional, making it challenging to quantify and distinguish differences among samples. PCA can reduce multidimensional data to a 2-dimensional coordinate system composed of 2 principal components, PC1 and PC2; then, distances in this 2-dimensional space can be used to assess differences between samples. When PCA fails to differentiate samples, methods such as partial least squares discriminant analysis (PLS-DA) and orthogonal projections to latent structures discriminant analysis (OPLS-DA) can be used to identify differences between groups on the basis of predefined grouping (Fig. 4E) [82,83]. The predictive ability of the OPLS-DA model is assessed through the predictive parameter *Q*^2^, with a *Q*^2^ value greater than 0.5 indicating reliable predictive power. Overfitting, suggesting a deviation from the original goals of prediction, occurs if the model fits the data too well. Permutation tests are conducted by randomly reshuffling the model data 500 times to check for overfitting (Fig. 4E). The model’s goodness of fit is assessed by analyzing the intercept of the regression line of *R*^2^ and the *Q*^2^ values on the *Y*-axis. If *R*^2^ and *Q*^2^ are both lower than the model’s predicted values, the model is considered free of fitting bias and reliable for further analysis. The scikit-learn package can be applied to perform multivariate analysis [84].

### Data analysis for spatial genomics methods

#### Preprocessing of genomics data

Preprocessing of spatial genomics data is similar to that of spatial transcriptomics methods based on spatial barcodes; however, a universal rapid analysis pipeline, such as SpaceRanger, is lacking. Consequently, genomic data processing involves sequence alignment and filtering, calculation of transcription start site (TSS) enrichment scores, matching of spatial barcodes, bead filtering, spatial binning, and normalization of genomic coverage. Postsequencing, the amount of raw data is substantial, with high-quality fastq files reaching sizes of up to 100 Gb. Therefore, fastq files can be split into separate files of 1 or 5 million reads each. Spatial barcodes are then extracted from each read and added to the sequence identifier column to facilitate subsequent alignment of each bead. Trimmomatic software can be used to remove Illumina adapters and low-quality short-read fragments [37]. Reads are aligned to the genome using STAR software and sorted by chromosome and mapping quality [38]. Unique molecular identifiers (UMIs) tools can then be used to merge reads corresponding to the same spatial barcode. TSS enrichment scores are calculated by dividing the read count at each position within 2,000 bp by the average read count between 1,800 bp and 2,000 bp. Bead barcodes are then mapped to the corresponding Illumina sequencing barcodes. Mapping accuracy is corrected on the basis of gene fragment length and distance. Beads that deviate from the array or do not cover the tissue are filtered out, and low-quality beads more than 1.6 mm from the center or with fewer than 10 aligned reads are discarded. The genome is divided into bins of varying sizes, with copy numbers determined by bin size. Beads are aggregated using the KNN technique, with the neighborhood diameter determined by the value of k. Each aligned read is assigned to a bin on the basis of its R1 genomic location and to a bead on the basis of its spatial barcode. A sparse count matrix is then created from these bin-bead pairs. To ensure a GC content greater than 35% and a mappability greater than 0.7, the data are normalized through local regression. Counts are also normalized by the replication timing and Tn5 insertion bias. The sparse count matrix is then divided by paired-end bulk sequencing data to adjust for batch effects in the samples. Finally, the genomic coverage of the diploid samples, bin size, and number of beads are assessed to evaluate the accuracy of the copy number analysis.

#### Clonal area

Compared with single-cell whole-genome sequencing techniques, slide-DNA-seq enables the visualization of clonal variations within tissue regions (Fig. 4F). To measure spatial variations in DNA copy numbers, the sparse count matrix for each array is smoothed using the KNN algorithm. The outlier bins are removed on the basis of the GC content, genomic location, and read count. The matrix data are subsequently subjected to PCA, and numerous PCs that explain an important proportion of the variance among the beads are selected and projected to the tissue regions. With this process, *n* distinct clonal differences within the tissue image are identified. The PCA result for each bead is then smoothed using the KNN algorithm, with the resulting scores used for k-means clustering. The Calinski–Harabasz index measures the tightness and separation of different clusters by comparing the variance associated with intracluster and intercluster dispersion of the beads, and the highest value from each clustering attempt is selected as the optimal k for k-means clustering. Finally, the clusters are projected onto tissue regions, completing the identification of different clonal areas. Zhao et al. [31] successfully identified 3 distinct clusters of genomic profiles in KrasG12D/+Trp53−/− mouse liver metastasis data, which may be used to study the evolutionary processes of in situ and metastatic tumors.

#### Enrichment analysis of the genomic region

Genomic region enrichment analysis reveals many genetic aberrations within tissue regions (Fig. 4F). In the preprocessing stage, the normalized matrix undergoes 500 permutations, ensuring that each bead in the tissue region is allocated the same number of reads. This allows for the calculation of empirical standard deviations within each region and for each bead within the background distribution created by the 500 permuted count matrices. Bead counts and permuted counts are spatially smoothed using the KNN algorithm. The observed bead counts are adjusted by subtracting the average counts assigned to pseudonormal clonal regions and then dividing by the standard deviation of the background distribution to calculate *z* scores and *P* values. The *z* scores of the nearest neighbors are then smoothed, facilitating the visualization of *z* score values within the tissue regions. To test whether specific genomic regions in different clusters have higher or lower coverage, a 2-sided Wilcoxon rank sum test can be applied, enabling the visualization of *P* values within the tissue regions of enriched genomic regions. Zhao et al. [31] reported amplifications of chr8q and deletions of chr15 and chr18, which are common across all tumor areas, suggesting that they appear early in tumor evolution and may play important roles in tumorigenesis.

#### Integrated spatial transcriptomics techniques

By integrating Slide-DNA-seq with Slide-RNA-seq, a series of subclone/tumor density-associated genes were identified (Fig. 4F). Enrichment analyses were conducted separately for Slide-DNA-seq and Slide-RNA-seq to determine tumor densities. To estimate local tumor and immune densities for each Slide-RNA-seq bead, the nearest neighbors in the *XY* space for each bead were identified. The local tumor and immune densities for each bead were then calculated on the basis of the percentage of these neighboring beads assigned to the tumor and immune clusters, respectively. The MATLAB function “imregister” was subsequently used to coregister the slices. Using the transformed matrix, clonal labels were transferred to spatial tumor clusters via Slide-RNA-seq. The average gene expression levels in each spatial tumor region were calculated. The average densities of the tumor and immune beads for each clone were then combined as variables in a fitted stepwise regression model to estimate the expression levels. The explained sum of squares (ESS) was used to calculate the percentage of variance that each variable contributed to explaining the model and the unexplained variance. Zhao et al. [31] identified genes associated with subclones, such as PLAG1, an oncogene located on chromosome 8q39, and MCM7, a MYC target gene on chromosome 7q involved in the initiation of DNA replication.

### Data analysis for spatial epigenomics methods

#### Data preprocessing

Deng et al. [29] designed a universal data preprocessing pipeline for Spatial-CUT&Tag and Spatial-ATAC-seq, which was released on the GitHub platform (https://github.com/dyxmvp/Spatial_ATAC-seq, https://github.com/dyxmvp/spatial-CUT-Tag). The preprocessing pipeline was developed via the Snakemake workflow management system. The core principle is to reformat the raw Fastq files to the Cell Ranger ATAC format (10x Genomics) [85]. The original raw sequencing files include read1 and read2 data. Through the BC_process.py script, the raw fastq files are reformatted as new read1 (containing the genome sequences), new read2 (containing spatial barcodes A and B), and new read3 data. After the organizational images are registered with sequencing chip images, each barcode in every sequence corresponds to a coordinate index provided by the sequencing chip. The resulting fastq files are aligned to the genome, filtered for duplicates, and counted via Cell Ranger ATAC, which generates fragment files for downstream analysis. Peaks in the Spatial-CUT&Tag data, which can be used to identify regions enriched with aligned reads in the genome, were found using MACs2 software [86].

#### Scoring models

The downstream data analysis is conducted via the ArchR [87] package and Seurat [39] package. Tissue chips are aligned with microscopy images to determine pixel locations. Adobe Illustrator software is used to select specific areas within the tissue. The Python scripts provided by Spatial-CUT&Tag and Spatial-ATAC-seq and the Seurat workflow are used to obtain the metadata files. The combined genomic matrix is then read with ArchR. Pixels that are not on the tissue are removed on the basis of the metadata files generated in the previous step. ArchR analysis is performed based on arrow files, with each arrow file storing all the information for a single sample. Within the ArchR package [87], data normalization and dimensionality reduction are performed via latent semantic indexing. Graph clustering and UMAP embedding are then carried out to successfully identify multiple groups of cell clusters in the images.

Scoring models can be employed to identify correlations between various biological functions and specific datasets, facilitating the experimental search for target biomarkers (Fig. 4G). In the ArchR package, the gene score model includes various computational models that generate gene score matrices for downstream analysis. Functions such as “getMarkerFeatures” and “getMarkers” in ArchR can be used to identify marker genes and regions. In the ArchR package, certain weights are applied to genes through the “addImputeWeights” function, mitigating the impact of gene length to some extent. If the parameter “addGeneScoreMat” is set to TRUE, gene scores are calculated during the creation of each arrow file, improving the analysis. Motif enrichment and motif deviations are calculated using the “peakAnnoEnrichment” and “addDeviationsMatrix” functions in ArchR.

#### Integration with scRNA-seq data

The cell types that were identified with this approach were similar to those identified with the spatial transcriptome deconvolution process (Fig. 4G). By integrating the reference scRNA-seq data with spatial genome data, cell-type recognition and pseudoscRNA-seq profiles can be obtained. The alignment of the gene expression matrix and the image was performed via the FindTransferAnchors function in the Seurat package. The cell annotation files and gene expression matrix were then imported into ArchR via the addGeneIntegrationMatrix function [87].

#### Pseudotemporal analysis

Pseudotemporal analysis revealed spatiotemporal changes in the chromatin accessibility of important genes (Fig. 4G). ChromVAR [88] can be used to identify transcription factor motifs for different cell types. The sparse chromatin accessibility data were analyzed by estimating the activation and inactivation of different genes. The analysis also revealed known and novel sequence motifs associated with changes in chromatin accessibility. Pseudotemporal reconstruction was implemented via the addTrajectory function in ArchR.

The current spatial epigenomic data analysis pipeline is designed for single-cell epigenomics. However, more spatial epigenomic data analysis pipelines that include extensive image processing and data integration should be developed.

### Combined with single-cell multiomics

Single-cell omics have the advantage of high throughput and high coverage, making them more suitable for discovering rare cell subtypes and exploring cellular heterogeneity. Spatial omics leverage their strength in retaining spatial information, which can reveal the spatial distribution of molecular expressions and the spatial interactions between cells

There are 2 main methods for integrating scRNA-seq and spatial data: deconvolution and mapping [46]. For more details, refer to the first “Identification of cell types” section. A spatial multiomics analysis integrates snRNA-seq, scATAC-seq, and spatial transcriptomics. By integrating snRNA-seq (single-nucleus RNA sequencing) and scATAC-seq (single-cell ATAC sequencing), and then using snRNA-seq deconvolution to score spots in spatial transcriptomics, Kuppe et al. [89] constructed gene regulatory networks that provide a comprehensive understanding of the cellular landscape and regulatory mechanisms within a tissue. Liu et al. developed the Spatial-CITE-seq multiomics technology, which integrates scCITE-seq with Spatial-CITE-seq data through the Seurat package, showing that these 2 datasets share highly consistent protein expression patterns [4].

Spatial multiomics will gradually become a technology that is compatible with high throughput, high resolution, and spatial localization.

## Data Analysis for Spatial Multiomics

**Fig. 5.**
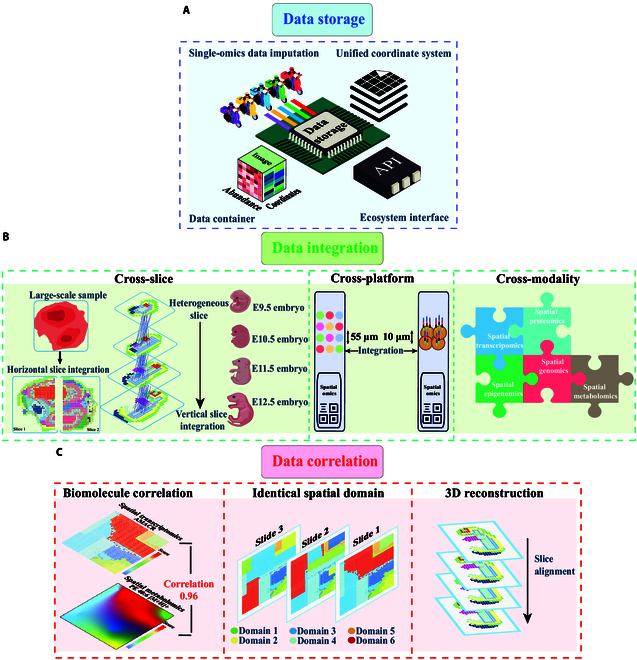
Spatial multiomics data analysis pipeline. (A) Data storage: A standardized framework for spatial multiomics data needs to undertake multiple tasks, including monoomics data imputation, data containers, a unified coordinate system, and an ecosystem interface. (B) Data integration: Based on the characteristics of multiomics technologies, data integration strategies can be categorized into cross-slice, cross-platform, and cross-modality. Depending on the heterogeneity of the slices, slice integration can be divided into horizontal slice integration and vertical slice integration. Different technical platforms exhibit varying spatial resolutions. Cross-platform factors must be considered during data integration. Cross-modality primarily integrates different single spatial monoomics methods. (C) Data correlation: Integrated spatial multiomics data can be used to explore biomolecular correlations, identify identical spatial domains, and perform 3D reconstruction.

Compared with the well-established single-cell multiomics field, spatial multiomics is still in the early stages of technological development, and established data analysis pipelines and integration strategies are lacking. However, many multiomics data analysis techniques are worth considering. Next, we focus on the data analysis pipeline and propose integration strategies suitable for spatial multiomics data (Fig. 5).

### Data storage

Spatial multiomics data are obtained by imputing monoomics data through different techniques and heterogeneous measurements. This process is complex, posing challenges for exploring biological sciences and understanding complex cellular states. To address these challenges, a standardized framework for storing diverse data formats, establishing unified coordinate systems, and creating interfaces linking multiple analysis tools is essential. Toolkits based on R and Python, such as Seurat [39], Giotto [40], and SpatialData [90], offer comprehensive frameworks for the management and analysis of spatial monoomics data (Fig. 5A).

The data storage framework must accommodate biomolecule abundance, image, and spatial coordinate information from a variety of technological platforms, enabling flexible data combination and transformation. The Seurat object [39] and Giotto object [40] in the R language platform create unique slots for each type of data in S4 format. The SpatialData object [90] in the Python language platform uses the zarr format to store datasets. These data storage containers are compatible with raw data from common commercial technologies, making it convenient for users to retrieve data through object formats. In addition, such frameworks should allow users to explore and analyze data across multiple dimensions and scales. The creation of a unified coordinate system is crucial. This system should align biomolecule coordinate information with image coordinate information to facilitate user queries on the spatial distribution patterns of the abundance levels of multiple biomolecules. This interface is key to streamlining multimodal analysis of spatial multiomics data, encompassing data preprocessing and downstream analysis.

### Data preprocessing

After importing multi-modal data into the data container, the methods for preprocessing and normalization primarily involve processing spatial multiomics data through spatial monoomics computational models. The main processes include filtering, normalization, dimensionality reduction, image processing, and handling of missing data. The processes of filtering, normalization, and dimensionality reduction are the same as the “Data Analysis Pipeline for Spatial Monoomics” section.

After processing molecular abundance data, it is necessary to proceed with cell segmentation and image registration for the image data. A variety of segmentation strategies are based on nuclear shape rules or DAPI staining marker strategies to segment cell nuclei. Various ML-based segmentation tools include Unet [91], Mesmer [92], and CellPose [93]. Recent methods like JSTA and SCS [94,95] define cell boundaries based on transcript distribution. Different commercial platforms offer their own alignment tools, such as Space Ranger and STCellBin [96].

The imputation of missing data is another crucial step, which involves using computational methods to infer missing molecular expression data at specific tissue locations. Various models based on scRNA-seq data are commonly used to impute spatial transcriptomic data, such as GimVI and Tangram [41,97]. Additionally, several missing value imputation methods are based on ML algorithms, such as the KNN imputation for mass spectrometry-based spatial proteomics [61].

### Data integration

Currently, various spatial monoomics technologies and analysis toolkits have been used for single-molecule analysis of different biological problems. To achieve multimodal integration of spatial multiomics data, it is necessary to design statistical models that fully consider the characteristics of different omics technologies and biomolecules. To overcome batch effects in the integration process of spatial multiomics data, different algorithmic models employ various strategies. In deep learning models (SLAT [98], STAligner [99], DeepST [100], and SPACEL [101]), the primary approach is to extract shared features and construct a joint latent space using Graph Autoencoder networks (GANs). Specifically, GANs create a cell adjacency matrix by extracting shared features from different batch datasets. Subsequently, the Joint Latent Space is constructed through the GAN algorithm. Within this joint latent space, comparisons and analyses are made between different sample pairs.

Researchers such as Argelaguet et al. [102] have proposed choosing different anchor points according to the experimental design to integrate different single-cell multiomics strategies (horizontal integration, vertical integration, and diagonal integration). However, this integration strategy may not be entirely suitable for spatial multiomics data, as spatial monoomics techniques have technical limitations and involve complex image information. Therefore, when devising integration strategies for spatial multiomics data, it is crucial to consider additional factors (Fig. 5B and Table 3).

**Table 3.** Packages for data integration

Package	Assumptions	Description	Model	Limitations and solutions	Ref.
SLAT	Cross-platform	Integrating datasets from different ST technologies: image-based ST (SeqFISH and Xenium) and barcode-based ST (Stereo-seq and Visium)	Graph neural networks and adversarial matching	Limitation 1: SLAT cannot account for local flexible distortions. Solution 1: Future work could develop a new module reference by B-spline Free-Form Deformation [144] to adjust local flexible distortions.	[98]
	Cross-modality	Integrating spatial transcriptomics (Stereo-seq) and spatial epigenomics (spatial-ATAC-seq)			
	Cross-slice	Vertical slice integration: Integrating spatial–temporal transitions slice: 4 mouse embryo slices at the time stages of E9.5, E10.5, E11.5, and E12.5 using Stereo-seq			
STAligner	Cross-platform	Integrating datasets from different resolution ST technologies: Slide-seqV2 with 10 μm resolution and Stereo-seq 220 nm resolution	Graph attention neural network	Limitation 1: STAligner, based on the Iterative Closest Point (ICP) algorithm, cannot account for local flexible distortions. Solution 1: Future work could develop a new module reference by B-spline Free-Form Deformation [144] to adjust local flexible distortions.	[99]
	Cross-slice	Vertical slice integration: Integrating heterogeneous slices from different samples: 12 squamous cell carcinoma slices from 4 patients using Visium and 4 mouse embryo slices at the different stage samples of E9.5, E10.5, E11.5 and E12.5 using Stereo-seq			
DeepST	Cross-platform	Integrating datasets from different resolution ST technologies: Visium with 55 μm resolution and Stereo-seq 220 nm resolution	Graph neural network	Limitation 1: Computational methods employing GNNs require large memory to load the entire graph. Solution 1: Optimizing memory efficiency through GNN mini-batch processing, parallel techniques, or even distributed learning systems is an important research direction. Limitation 2: The model’s applicability and convergence stability need further consideration due to performance disparities in unsupervised deep learning method. Solution 2: Future work should use random seeds to control convergence.	[100]
STACI	Cross-modality	Integrating spatial transcriptomics and chromatin images	Graph convolutional network	Limitation 1: The STACI model may require a large number of parameters and computational resources. Solution 1: leveraging distributed computing resources to handle large datasets could be considered. Limitation 1: The STACI model may have limitations in generalizability when dealing with data from different tissues or disease states. Solution 1: Improving the model’s generalizability could be achieved by training and validating on a diverse range of datasets.	[104]
SMOx	Cross-modality	Integrating spatial transcriptomics (Visium) and spatial metabolomics (MALDI-MSI)	Co-registration and granularity matching	Limitation 1: SMOx cannot account for local flexible distortions. Solution 1: Future work could develop a new module reference by B-spline Free-Form Deformation [144] to adjust local flexible distortions. Limitation 2: Batch effects can restrict the comparison of multiple samples. Solution 2: It highlights the need for further method development to mitigate the effects of batch effects.	[105]
PRECAST	Cross-slice	Vertical slice integration: Integrating heterogeneous slices from different samples: 12 human dorsolateral prefrontal cortex slices from 3 donors using Visium. Horizontal slice integration: Integrating adjacent slices: hepatocellular carcinoma from tumor region and tumor-adjacent region using Visium	Principled probabilistic model	Limitation 1: PRECAST does not employ additional algorithms to address both rigid and non-rigid transformations in images. Solution 1: In future work, it needs address rigid transformation issues through manual or automated algorithms and adjust non-rigid transformations using B-spline Free-Form Deformation [144].	[103]
SPACEL	Cross-slice	Vertical slice integration: Integrating heterogeneous slices from different samples: 12 squamous cell carcinoma slices from 4 patients using Visium and 11 human breast cancer slices from 11 samples.	Graph convolutional network	Limitation 1: When adding new slices to the dataset, the entire deep learning model needs to be retrained from scratch. Solution 1: The future improvements could involve implementing transfer learning techniques to enhance the efficiency of SPACEL when analyzing large-scale datasets.	[101]

Cross-platform strategies: Omics platforms have different resolutions. Among barcode-based methods, the resolution of Visium [6] is 55 μm, and the resolution of Stereo-seq [5] is further improved to the subcellular level (Fig. 5B). Therefore, it is necessary to set common anchor points in the model to integrate data collected with different resolutions. It is difficult to merge spatial monoomics data collected by different platforms because of the varying amounts of intracellular information, use of spatial coordinate systems, and batch effects. DeepST [100] calculates distances between spots using spatial coordinates and constructs a cell–cell spatial relationship graph based on the top nearest neighbors. These are then integrated into the Slide-seqV2 and Stereo-seq platforms. In SLAT [98], a multimodality embedding strategy that aligns heterogeneous spatial data by using different neighborhood sizes for mapping on the basis of the resolutions of the technologies is used. SLAT allows for the selection of a different number of neighbors (k value) for each section to accommodate its spatial resolution. SLAT aligns image-based spatial transcriptomics data (Visium and Xenium) with barcode-based spatial transcriptomics data. STAligner [99] learns spatially aware embeddings based on a spatial adjacency graph. These embeddings take into account spatial coordinates and gene expression data, allowing the model to integrate data of varying resolutions by focusing on spatial relationships rather than the differences in resolution. STAligner was used to identify common and unique tissue structures in mouse olfactory bulb slices obtained from 2 different platforms (Slide-seq2 and Stereo-seq).

Cross-slice strategies: Owing to the technical limitations of the sequencing platform, the capture area of spatial monoomics technology (for example, the capture area of Visium [6] is 6.5 mm × 6.5 mm) may be smaller than the tissue sample area. Therefore, it is necessary to use an integration model to combine multidimensional data in adjacent slices of the same sample or different samples. On the basis of the degree of slice heterogeneity, slice integration can be classified as vertical slice integration and horizontal slice integration (Fig. 5B). Vertical slice integration methods involve combining slices from different samples, whereas horizontal slice integration methods involve combining different regions within the same slice (Fig. 5B). PRECAST [103] uses a unified and principled probabilistic model to eliminate batch effects from samples and employs cell/domain labels for precise vertical and horizontal slice integration. In STAligner [99], the concept of triplets is introduced, with batch differences minimized through triplet loss-based correction of distances between different anchors and iterative optimization via autoencoder training. With this method, horizontal slices of 12 squamous cell carcinoma slices from 4 patients and 4 mouse embryo slices at different stages were integrated. SPACEL [101] employs graph convolutional networks and adversarial learning algorithms, and this method was used to vertically integrate 12 squamous cell carcinoma slices and 11 human breast cancer slices. SLAT [98] uses graph neural networks and adversarial graph alignment, and this method was used to stably align 4 mouse embryo slices at the E9.5, E10.5, and E11.5 stages.

Cross-modality strategies: Spatial monoomics techniques, such as DBiT-seq [17], which can simultaneously detect mRNAs and proteins (Fig. 5B), have developed from monomics analyses within sections to spatial multiomics. To understand the correlation between different omics levels, it is necessary to calculate the correlation between different biomolecules with an integration model. STACI [104] is the first method to integrate all available modalities simultaneously, including gene expression, CN, and chromatin imaging approaches. It can translate between different data modalities and has been used to identify the spatiotemporal progression of Alzheimer’s disease. SMOx [105] jointly registers Visium and MALDI-MSI tissue images. Gaussian weighting is used to address cross-platform resolution issues. This method has been used to explore spatial correlations of transcripts and lipids within a single patient’s tissue slice. SLAT [98] uses a lightweight graph convolutional network to align tissue images collected with Stereo-seq and Spatial-ATAC-seq. This allows for the transfer of cell-type labels and ensures consistency between tissue-specific genes and chromatin.

Multiple computational model integration strategies, including cross-platform, cross-slice, and cross-modality strategies, are discussed in terms of their assumptions, limitations, and solutions when integrating spatial multiomics data. Issues such as the inability to handle flexible transformations, computational efficiency, and challenges with large datasets are addressed. These issues and their solutions are listed in Table 3.

### Data correlations

#### Biomolecule correlations

Organically integrating spatial multiomics data and constructing regulatory networks between molecules can aid in comprehensively exploring and understanding the regulatory and causal relationships between biological molecules. This approach allows for the accurate interpretation of biological functions and physiological mechanisms in living organisms (Fig. 5C). To match the granularity of spatial resolution, SMOx [105] applies Gaussian weighting to MSI pixels near each ST spot after registration. A labeling system is used to align snRNA-seq and MSI data across modalities, which has been used in analyses of prostate cancer. MSMB-PE 40:2 [M+H]+ was used as the tumor-specific gene–lipid pair. Owing to the joint latent space providing a combined characterization of spatial transcriptomics data and chromatin images, a STACI [104] trained model then incorporates new chromatin imaging data to predict spatial transcriptomics data. STACI predicts the size of amyloid plaques near different cell types (defined by spatial transcriptomics data) through chromatin imaging, thereby monitoring the progression of Alzheimer’s disease. SLAT [98] projects cells from different modalities into a shared embedding space before inputting them into a lightweight graph-convolutional network. Through this expansion, SLAT successfully generates spatial alignment across distinct modalities. In addition, in this study, SLAT identified the chromatin accessibility score and gene expression pattern of the heart marker Tnnt2. SCENIC+ enables the integration of Spatial-ATAC-seq, scATAC-seq, and scRNA-seq data, which can be used to predict genomic enhancers and candidate upstream transcription factors and connect these enhancers with candidate target genes [106].

#### Identical spatial domains

Cells specifically aggregate in space to form repeated functional units, which are closely related to specific biological functions. With the development of spatial monoomics technology, researchers have developed a variety of computational methods to identify spatial domains in tissues. However, in cross-slice and cross-platform data integration, most spatial monoomics data collected in different tissue slices cannot be processed due to batch variations (Fig. 5C). To address the above challenges, researchers have developed new methods for identifying spatial domains. In DeepST [100], the spatial information of spatial transcriptomics data is used to construct neighborhood graphs, reconstruct gene expression matrices and identify the same spatial domains across different samples. In one application of DeepST, MERFISH data from 3 consecutive batches of mouse preoptic hypothalamus samples were integrated, and the 3-dimensional structural domains of “Ependymal” and “OD Mature” were clearly resolved. In STAligner [99], batch effects are reduced by minimizing the triplet loss and autoencoder reconstruction loss while identifying tissue structures with similar spatial expression patterns. STAligner effectively captures shared spatial domains across different slices, Alzheimer’s disease-related substructures, and dynamic changes during mouse embryo development. The probability model-based PRECAST [103] simultaneously performs spatial feature extraction, spatial clustering, and low-dimensional alignment to obtain similar spatial domains across multiple tissue slices. In one application of PRECAST, 4 sections of hepatocellular carcinoma tissue were integrated, and a tumor/normal epithelium region that is shared across multiple sections was identified.

Additionally, enrichment analysis offers more evaluations of biological functions and cell types within the spatial domain. Enrichment analysis facilitates a deeper exploration of biological functions and processes within spatial domains or cell types. Common scoring methods for functional sets include GSEA [107], GSVA [108], and AUCell [109]. Various databases provide target datasets for scoring methods: GO [110], KEGG [111], ImmuneSigDB [112], and MSigDB [113].

#### 3D reconstruction

Biological tissues and organs develop in 3-dimensional space, such as through the development of embryos into complete organs and individuals. However, having only 2-dimensional tissue information greatly limits our ability to analyze the process of biological signal transmission in space and how cells interact with each other. Spatial monoomics methods can be used to successfully associate 2-dimensional tissue images with biomolecule information, providing opportunities to explore 3-dimensional tissue structures.

Recently, various researchers have used 3-dimensional reconstruction methods to align and register multiple slices to reconstruct 3-dimensional tissue structures (Fig. 5C). STAligner [99] employs the mutual nearest neighbors (MNN) algorithm to assess anchor points for the alignment of adjacent sections, followed by the use of the iterative closest point algorithm to achieve rigid transformation. STAligner uses the spatial domain as a landmark to guide the registration process. Unlike existing tools, SPACEL adopts an MNN graph and the differential evolution algorithm to transform slices and construct a stacked 3D alignment of adjacent tissues [101]. In terms of 3-dimensional reconstruction algorithms, SPACEL outperforms STAligner. A higher Pearson’s correlation coefficient and structural similarity index were achieved when the transformed coordinate system and the original coordinate system were evaluated.

### Validation metrics

We recommend evaluating the methods’ performances in data integration, obtaining embeddings for cellular biological effects, and spatial clustering using the following metrics: To assess performance in batch-effect removal, we used the cell-type/integration local inverse Simpson’s index (LISI), cell-type LISI, and integration LISI [114]. The F1-score can be used to indicate the effect of preserving biological variation after integrating datasets. The CCor is the mean canonical correlation between the estimated features and the true ones [115]. The Concr measure is the mean correlation between gene expression and cell type [115]. Additionally, to evaluate the similarity between different modalities or sections in spatial domains, the adjusted Rand index (ARI) measures the similarity between 2 different domains [103].

## Application of Spatial Monoomics Techniques

### Tumor

**Fig. 6.**
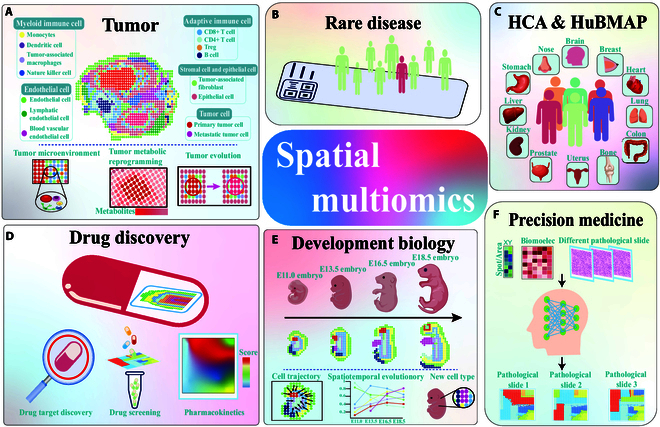
Application of spatial monoomics. Spatial multiomics technologies have been applied in cutting-edge research across 7 fields. (A) In oncology, spatial multiomics technologies are used to explore the tumor microenvironment, tumor metabolic reprogramming, and tumor evolution. (B) These technologies are employed to uncover the hidden mechanisms of rare diseases. (C) Spatial multiomics has deepened our understanding of the human body’s structure and organ composition. (D) Spatial monoomics is used for drug target discovery, drug screening, and pharmacokinetic exploration. (E) Spatial monoomics precisely defines cellular functional states and developmental trajectories and captures the spatiotemporal evolution of cells. (F) When combined with AI, spatial multiomics can assist in the diagnosis of pathological slices.

A tumor is a dynamically evolving entity. The TME is composed of tumor cells, immune cells, stromal cells, fibroblasts, the extracellular matrix, and blood vessels (Fig. 6A) [116]. To adapt to competition for metabolites and nutrients, therapeutic pressure, or the evolution of key oncogenes, the properties of the TME are constantly evolving. Spatial multiomics technologies (including genomics, epigenomics, transcriptomics, proteomics, and metabolomics) can integrate biological molecules and their physical spatial locations, which may systematically advance tumor research (Fig. 6A).

The cellular composition and functional state of the TME can vary greatly depending on the organ of tumor origin, intrinsic characteristics of the tumor cells, stage of the tumor, and specific features of the patient (Fig. 6A). Cancer-associated fibroblasts (CAFs) interact directly with tumor cells, reshaping the extracellular matrix (ECM) and suppressing immune cells through the release of cytokines, exosomes, and nutrients [117]. In breast cancer, immune-infiltrated iCAFs are distributed in areas containing both tumor cells and immune stromal cells, whereas myofibroblast CAFs that regulate the ECM are predominantly found in tumor cell-rich areas [118]. Moncada et al. [119] reported that tumors recruit CAFs to form spatial functional units that serve specific functions.

During the development and progression of tumors, the complexity and dynamic interplay among components within the TME lead to important changes in the metabolic landscape (Fig. 6A). Sun et al. [120] constructed a metabolome–transcriptome correlation network map for gastric cancer that was colocalized within heterogeneous cancer tissues. The peritumoral and distal lymphoid tissues in the tumor interface region presented important differences in glutamine and fatty acid-related metabolites, clearly indicating metabolic reprogramming in the gastric cancer microenvironment. Risom et al. [121] used MIBI technology to construct spatial maps of ductal carcinoma in situ (DCIS) and invasive breast cancer. They observed that DCIS has a higher presence of myoepithelium, stromal hypertrophic cells, and CD4T cells.

At the single-cell level, individual cells evolve to show distinct subclonal patterns through multiple rounds of division, mutation, and selection (Fig. 6A). Without the constraints of histological boundaries, spatial genomics can provide important insights into the clonal architecture of tumors and adjacent benign tissue. Erickson [122] developed a tool for predicting copy number variations (CNVs) using spatial transcriptomics, siCNV, which has been used to map the clonal landscapes of prostate tumors and neighboring benign tissues and to elucidate the relationships between clonal subtypes and histological classifications.

### Rare diseases

Rare diseases pose substantial challenges for global public health (Fig. 6B). Currently, the mechanisms underlying many rare diseases remain unclear, and treatments are largely ineffective and are associated with a high risk of complications. However, with the advancement of spatial multiomics technologies, potential solutions to these issues are being investigated. Idiopathic multicentric Castleman disease (iMCD) is a rare cytokine-driven disorder linked to genomic alterations at the IL-6/IL-6R loci [123]. Chan et al. [124] employed Stereo-seq and scRNA-seq techniques to analyze lymphatic tissues and peripheral blood samples from a pair of twins with iMCD, confirming that functional alterations in NCOA4 and TRAF3 lead to the onset of iMCD. In addition, hereditary sensory and autonomic neuropathy 9 (HSAN9) is a rare and fatal neurological disorder caused by mis- and nonsense mutations in the gene encoding Tectonin β-propeller repeat-containing protein 2 (TECPR2 ) [125]. Nalbach et al. [126] used spatial proteomics techniques to determine that HSAN9-truncated TECPR2 impacts various proteins related to neuronal function via intracellular transport. In clinical and laboratory settings, exome sequencing and whole-genome sequencing techniques are employed to screen for rare diseases. Spatial multiomics techniques enable the acquisition of comprehensive biomolecule and tissue spatial information for rare diseases. However, the heterogeneity of tissues and the instability of spatial monoomics technologies are still limitations in clinical applications. Tissue invasiveness, high costs, and lengthy testing cycles are also important factors to consider for patients.

### The HCA and HuBMAP

With the advancement of multiomics technologies, initiatives such as the HCA and the HuBMAP have been launched (Fig. 6C) [1,2]. Both of these initiatives aim to establish spatial multiomics maps of nondiseased human organs as a foundation for understanding human health and for diagnosing, monitoring, and treating diseases. Spatial multiomics techniques are gradually becoming the most suitable and optimal tools for accomplishing these tasks.

The development of spatial multiomics technologies has enabled the exploration of intercellular biomolecule mechanisms and the construction of 3-dimensional maps of the human body and organs. For example, the secondary lymphoid organs (SLOs) are crucial immune organs in humans. Massoni-Badosa et al. [127] explored immune cell activation in SLOs and identified an unreported transcription factor, SIX5, as a marker of mature plasma cells that potentially plays a role in regulating multiple myeloma tumorigenesis. In addition, kidney diseases affect more than 10% of the population worldwide, and many gaps in research remain regarding the specific functions of different cells within the kidney. Li et al. [128] mapped the transcriptional, epigenetic, and metabolomic landscape of 48 adult kidneys and identified distinct transcriptomic, chromatin accessibility, and metabolomic signatures in different regions of the kidney, such as the thick ascending limb of the loop of Henle, principal cells, and thin limb.

### Drug discovery

Drug target discovery, drug screening, and pharmacokinetics have long been important tasks in medical engineering. Spatial genomics, with its unique characteristics, plays a vital role in drug development (Fig. 6D). Spatial genomics techniques can be used to analyze unique gene expression patterns within target areas with single-cell spatial resolution, enabling the accurate identification of drug action targets. In genetically engineered organoids derived from GAN mice, researchers discovered that MAPK signaling promotes the development of tumors and that the TME is at the leading edge of tumor invasion [129]. In addition, spatial monoomics facilitates the screening of specific targets to test the combined effects of drugs [130]. In a study based on pancreatic ductal adenocarcinoma organoid models, the heterogeneity of the tumor cell composition was characterized, which revealed that ataluren can target GCNT3 for mucin regulation and enhance the antitumor effects of chemotherapeutic drugs when combined with gemcitabine and nab-paclitaxel [131]. In cancer drug development and pharmacology, the aim of spatial metabolomics can be summarized as identifying “the right drug in the right place at the right concentration”. For example, in a clinical trial, researchers measured the drug distribution of rifampin and rifapentine in a rabbit model of tuberculosis and reported that rifampin better penetrated cavitary lesions than rifapentine did [132].

### Development biology

Technical limitations restrict the ability of biologists to investigate key developmental events in human embryos. Breakthroughs in spatial monoomics technology may enable “black box” investigations in developmental biology. Spatial monoomics techniques can be used to precisely define cellular functional states and developmental trajectories and capture the spatiotemporal evolution of cells (Fig. 6E).

The human lung, a vital organ that begins functioning after birth, begins to develop long before birth. Mapping embryonic and fetal human lungs can assist in the identification of precursor and progenitor cells. He et al. [133] conducted deep sampling of lung samples from 5 to 22 PCW and identified 2 types of NE cells: GRP+ NE cells and GHRL+ NE cells. N-type human small cell lung cancer cells are GHRL+ NE cells. The heart, the first solid organ to develop in the human embryo, undergoes the formation of 4 independent chambers, after which the outflow tract differentiates into the aorta and pulmonary artery. Asp et al. [134] were the first to reveal unique gene expression patterns in different anatomical regions of the human embryonic heart at 4.5 to 5, 6.5, and 9 PCW.

In mouse embryology, multiomics technologies are widely used. For example, Jiang et al. [135] employed spatial multiomics techniques to simultaneously capture ATAC and RNA data and analyzed mouse brain development at E11.0, E13.5, E15.5, and E18.5. They mapped the spatiotemporal trajectories of these data during cortical development and identified a regulatory cascade involving Pax6-Eomes-Tbr1.

These studies demonstrate that spatial multiomics methods are applicable not only for exploring biomolecule expression trends in single cells but also for tracking the spatiotemporal progression of tissue development.

### Precision medicine

Currently, clinical diagnostic strategies rely heavily on the experience level of clinical pathologists, and evaluation systems must be further optimized. With the advancement of deep learning and ML algorithms for gene-based studies, artificial intelligence (AI) techniques have been used to assist clinical pathologists in making decisive clinical diagnoses on the basis of pathological slides (Fig. 6F) [136,137]. In addition, researchers have employed ML to precisely delineate tumor regions. For example, Yoosuf et al. [138] trained a ML model based on breast cancer tissue sections and classified breast cancer regions on the basis of transcriptomic information.

Spatial monoomics methods have been applied in continuous spatiotemporal analyses of embryos at different stages, suggesting the feasibility of such techniques in monitoring disease progression in clinical settings. The Spatial iTracer technology combines spatial transcriptomics with genetic label-based lineage tracing to uncover the clonality of cell fates in human cerebral organoids [139]. Heiser et al. [140] deduce the dynamic evolution of subclonal tumors based on genetic events and places clonal regions along global pseudotemporal progression trajectories. However, the difficulty of continuous sampling in diagnostic and treatment plans in clinical settings must be considered. Studies based on hematoxylin and eosin (H&E)-stained sections and scRNA-seq abundance data have demonstrated the high diagnostic accuracy of existing techniques. However, more biomolecule information, such as protein, metabolite, and chromatin information, could be used to improve performance. Spatial-CITE-seq can combine conventional diagnostic markers (such as TTF1, napsin, p63, and cytokeratin) with other immune markers (such as PD-L1, PD-1, CD68, CD45, CD8, and CD3) to determine the optimal strategy for immunotherapy [4]. Phillips et al. [141] developed the SpatialScore, which is based on the distance between CD4+ T cells and the nearest cancer cells, to predict treatment responses in cutaneous T-cell lymphoma. This method has been shown to be more effective in predicting patients’ responses to pembrolizumab than traditional biomarkers [142].

## Future Work

With rapid development of AI, many deep learning approaches have been proposed [143]. AI methods can be used to comprehensively analyze tissue structures on the basis of interpretations of spatial multiomics data. Feature selection methods based on deep learning can be used to identify nonlinear data and interacting features. Pretrained deep neural network models that extract feature vectors from images, in conjunction with biomolecular abundance information, can be used to identify rare cell types and special anatomical structures within the spatial domain. The greatest challenge in integrating spatial multiomics data is the heterogeneity of the data, which is amplified when multiple types of data are considered. AI methods have been employed to correct for batch effects in single-cell multiomics data. The introduction of various types of tissue image information in spatial monoomics, such as mass spectrometry images, H&E images, and multiplex immunofluorescence images, complicates this issue. However, ML or deep learning techniques can be used to segment cells or extract feature vectors, which may enable the integration of heterogeneous data. AI/ML utilize deep learning models to select features of cells or spots within a shared joint latent space. Subsequently, clustering algorithms are applied to the extracted features to identify spatial domains. For sequential tissue sections, the aligned spatial domains are integrated to construct a 3D architecture of the tumor tissue, revealing the direction of tumor progression.

The developed AI models extend spatial holographic techniques to the field of clinical pathology. The differential distribution of biomolecules such as genes, proteins, and small-molecule metabolites leads to different tissue contours after staining. Therefore, the integration of heterogeneous slides and spatial monoomics through deep learning has great potential in localizing tumor positions, improving our understanding of tumor progression, and identifying therapeutic targets.

The aforementioned computational models employ differential evolution algorithms, adaptive cropping strategies, and ICP algorithms to calibrate rigid transformations, yet they overlook the issue of local deformations in images [98,99,101]. The GCN-based CAST [144] may offer an excellent example. CAST Stack utilizes the B-spline Free-Form Deformation method to address local deformations. By generating a grid on the spatial section and applying deformations to the control points of the grid, this approach allows for the warping of local areas while maintaining the overall structure, to better align different samples.

Single-cell multiomics, as a pioneer in the field of multiomics, is characterized by high coverage and high sensitivity. Integrating single-cell multiomics fully with spatial multiomics could overcome the limitations of low throughput in spatial multiomics and the lack of spatial location in single-cell multiomics. For instance, the combination of Spatial-CUT&Tag [27] with scCUT&Tag [145] can predict histone loss of expression in spatial multiomics. If scCUT&Tag-pro [146] is integrated with histone or protein data from spatial multiomics, it can describe protein–DNA interactions in space.

Current tools are no longer sufficient to meet researchers’ demands for data visualization. Researchers require a high-efficiency visualization tool that can interact directly with data, describe 3-dimensional reconstructed profiles, and colocalize multimodal data. Future visualization tools, with their superior functionality and user-friendly interfaces, will provide a new solution for the analysis of spatial multiomics data.

The application of spatial multiomics in clinical practice is still limited by stability, reproducibility, and high costs. The RNA within FFPE samples is susceptible to fragmentation during the paraffin-embedding process and may further experience heightened degradation under suboptimal storage [147]. Additionally, the chemical modifications that RNA may undergo and the loss of poly-A tails in the samples both limit the spatial multiomics options compatible with FFPE samples. First, multiomics instruments should be compatible with FFPE samples commonly used in clinical settings, which is a challenge that is currently in the research and development phase. Secondly, due to the constraints of high costs, current low-throughput assays meet basic clinical needs. High-throughput assays are ideal, but the high total cost of instruments, antibody costs, and experimental costs must still be considered. To our knowledge, spatial transcriptomics and spatial proteomics can cost thousands of dollars per sample. To better apply to early disease screening and monitoring, customized data analysis processes should be developed for different diseases, especially heterogeneous tumors. This requires considering tissue types, disease progression, and personalized factors during data analysis. Therefore, if spatial multiomics is to provide reliable diagnostic results for clinical use, a standardized data analysis workflow is crucial.

## Conclusions

In this work, we discussed recent advancements in spatial monoomics and spatial multiomics techniques, including technology, data analysis pipeline, and translational applications. We emphasize the importance of data analysis, providing a comprehensive summary of the data analysis pipeline for spatial monoomics data, from data preprocessing to downstream analysis. Although spatial monoomics techniques have become increasingly mature, the development of spatial multiomics approaches has transformed the field of spatial monoomics. However, the lack of spatial multiomics data analysis strategies has limited research in broader fields. Therefore, we propose several integration strategies and outline a complete multiomics data analysis pipeline.

## Data Availability

All data related to this paper is shown in figures and tables.
